# A Contrast Frugal Approach to Transcatheter Aortic Valve Replacement in Chronic Kidney Disease: A Pilot Study

**DOI:** 10.7759/cureus.32878

**Published:** 2022-12-23

**Authors:** Subhasish Bose, Brinder Kanda, Sasmit Roy, Kenneth Saum, John Haas, Fadi El-Adhab, Crystal Ranson, Nichole Brunton, Reagan Morford, Houman Tavaf-Motamen

**Affiliations:** 1 Internal Medicine, Liberty University College of Osteopathic Medicine, Lynchburg, USA; 2 Nephrology, University of Virginia, Lynchburg, USA; 3 Nephrology/Internal Medicine, Lynchburg General Hospital, Lynchburg, USA; 4 Internal Medicine, Liberty University College of Osreopathic Medicine, Lynchburg, USA; 5 Cardiology, Centra Lynchburg General Hospital, Lynchburg, USA; 6 Cardiovascular Surgery, CMG Stroobant's Cardiovascular Center, Lynchburg, USA; 7 Nephrology, Centra Lynchburg General Hospital, Lynchburg, USA; 8 Cardiovascular Surgery, Centra Lunchburg General Hospital, Lynchburg, USA; 9 Cardiovascular Surgery, Centra Lynchburg General Hospital, Lynchburg, USA; 10 Cardiovascular Disease, Centra Lynchburg General Hospital, Lynchburg, USA; 11 Internal Medicine, Danbury Hospital, Danbury, USA; 12 Science and Technology, Central Virginia Governor's School, Lynchburg, USA

**Keywords:** aortic stenosis, transcatheter aortic valve replacement, acute kidney injury, contrast induced nephropathy, chronic kidney disease

## Abstract

Background: Transcatheter aortic valve replacement (TAVR) is now regarded as a viable treatment option for all cases of severe aortic stenosis (AS). Acute kidney injury (AKI) is common and lowers the survival of patients after TAVR and iodine-based contrast-induced nephropathy (CIN) plays a significant adverse role in AKI. Therefore, in chronic kidney disease (CKD) patients requiring pre-operative evaluation for TAVR, the risk of CIN is of particular concern.

Methods: It was a single-center study including eight CKD patients who underwent pre-operative evaluation for TAVR with minimized contrast exposure by means of pre-operative contrast-sparing evaluation and intra-operative contrast minimization. All patients had glomerular filtration rate (eGFR) calculated before TAVR and on a follow-up about one month and one year post-operatively to document the impact of this TAVR protocol on prognosis of kidney function in patients with advanced CKD.

Results: New York Heart Association (NYHA) functional classification demonstrated significant improvement of symptomatology (p = 0.0001) by one-year post-TAVR. Patients’ mean AS gradient was significantly improved (p = 0.00004) after the TAVR procedure. No significant post-operative paravalvular aortic regurgitation was noted on follow up echocardiogram. eGFR data showed mean eGFR for the group was slightly better (27.38 ml/min/per 1.73 m^2 ^BSA vs. 30.38 ml/min/per 1.73 m^2 ^BSA) after TAVR.

Conclusions: “Contrast frugal” approach is feasible and safe for pre-TAVR evaluation and the procedure itself. Our pilot study showed no significant paravalvular leak of the prosthetic valve following this proposed protocol. No statistically significant decrease in eGFR was noted on a one-year follow-up.

## Introduction

Transcatheter aortic valve replacement (TAVR) has now emerged as a viable treatment option for all cases of severe aortic stenosis (AS), including patients who are considered otherwise low risk for surgical aortic valve replacement (SAVR) [1]. Despite encouraging published outcomes, acute kidney injury (AKI) is common and lowers the survival of patients after TAVR [2,3]. The pathogenesis of AKI after TAVR is multifactorial including TAVR-specific factors such as the use of iodine-based intravenous contrast dye agents, hypotension during rapid pacing, and embolization; preventive measures may include pre-procedural hydration, limitation of contrast dye exposure, and avoidance of intraprocedural hypotension. In recent years, the number of TAVRs performed worldwide has been increasing, as well as published data on renal perspectives of TAVR. The TAVR is a complex procedure, and its timely planning is crucial. If not planned appropriately, there are major complications that can occur, even mortality, e.g., significant paravalvular leak, valve embolization, annular rupture, coronary occlusion, conduction disturbance, etc. Even rare complications like strut inversion in the background of calcium spur have been reported [4]. The purpose of this paper is to establish the concept of contrast frugal technique that can be done safely without any significant complication. 

Prevalence of AS increases with age: at the rate of 1.3% in patients between 65 and 74 years, and 2.8% to 4.6% in patients >75 years of age [5,6]. The number of patients with AS in the United States continues to rise over time due to the aging population making AS a significant healthcare burden [6,7]. Without treatment, these patients have a poor prognosis with 50% mortality in the first two years after diagnosis [8]. In the past, SAVR used to be considered the gold standard treatment for severe symptomatic AS [9,10]. The TAVR has now established itself as an effective alternative to traditional SAVR irrespective of the patient’s risk profile or co-morbidities provided there is no technical contraindication for TAVR [1,11-13]. Typically, previous clinical TAVR trials have excluded advanced chronic kidney disease (CKD) patients; however, when comparing both TAVR and SAVR in this population, TAVR may be preferable for less likelihood of hemodynamic instability, peri-anesthetic complications, and post-surgical complications. Nevertheless, AKI remains an important post-SAVR and post-TAVR complication, particularly in patients with high comorbidities [14]. The preoperative presence of CKD has been identified as an independent risk factor for developing AKI and is associated with increased length of stay in the hospital and mortality [15,16].

The standard pre-operative workup for TAVR is extensive and involves a significant amount of intravenous iodine-based contrast dye (IVD) use. Using contrast may further deteriorate the renal function of patients with pre-existing advanced CKD. To improve the usefulness of the TAVR procedure in advanced CKD patients far beyond SAVR, minimizing the risk of worsening renal function may play a vital role and may translate into improved mortality and morbidity as CKD has been established as an important risk factor of poor outcome. We hereby present a modified pre-procedure evaluation strategy for TAVR that involves minimal exposure to IVD in a small case series of TAVR cases from our center.

Background

TAVR Trials

The most commonly discussed TAVR trials were conducted by the placement of aortic transcatheter valves (PARTNER) investigators. In 2010, data from this multicenter, randomized clinical trial showed the significance of transcatheter aortic-valve implantation (TAVI) for aortic stenosis in patients who cannot undergo surgery [17]. This study demonstrated decreased mortality and repeat hospitalization at one year in patients undergoing TAVR compared to patients undergoing medical therapy alone. The PARTNER investigators in subsequent publications investigated the role of TAVR as a promising option for high-risk surgical patients requiring SAVR. This trial demonstrated non-inferiority to SAVR and a 2% decrease in all-cause mortality in TAVR patients at one year compared to SAVR [18]. An additional trial named PARTNER 2, published in 2016, further supported the non-inferiority of TAVR to SAVR in intermediate surgical risk patients [19].

The TAVR as a procedure has been evolving to become the alternative standard of care for patients with severe AS. However, patients with CKD remain a challenge in the context of work-up and perioperative risks. The PARTNER cohorts A and B excluded patients with creatinine >3.0 or those undergoing renal replacement therapy (RRT) [17,18]. Similarly, the CoreValve trial excluded patients with end-stage renal disease or a creatinine clearance of <20 cc/minute, due to the need for contrast administration [20]. However, it is well recognized that TAVR outcomes are significantly related to pre-procedural kidney function and changes in kidney function after TAVR have a significant impact on mortality. The AKI after TAVR is associated with increased morbidity and mortality [21].

TAVR in Patients With CKD

There have been studies focused on the incidence of RRT following TAVR and the association of CKD with TAVR outcomes. One retrospective study utilized the Centers for Medicare and Medicaid database to identify patients who underwent TAVR between November 2011 and September 2015 [22]. The study evaluated the incidence of RRT following TAVR, in this population. A significant association of increased mortality was found in patients with low pre-procedural glomerular filtration rate (GFR) (<60 ml/min/㎡) and in patients requiring new RRT following TAVR [22]. Another study demonstrated that increased mortality was associated with new dialysis following TAVR but it also reported a decreasing proportion of TAVR patients requiring dialysis post-procedure. This proportion decreased from 6.1% between 2007 and 2008 to 2.3% in 2013 and 2014. Additionally, the study reported that the risk of new dialysis was found to be independently associated with moderate-to-severe aortic regurgitation post-procedure, the year of the procedure, lower baseline renal function, and diabetes [23].

Overall, patients undergoing the TAVR procedure have a higher risk of post-procedural complications, morbidity (AKI and need for RRT), and mortality in cases of advanced pre-procedural CKD. Unfortunately, there has been no noteworthy research on how to minimize the decline of renal function in CKD patients requiring TAVR procedures.

Current Standard Pre-procedural Work-Up Guidelines for TAVR

The current guidelines provided by the 2017 Expert Consensus for TAVR recommend three primary imaging modalities for patients preoperatively: coronary angiography, electrocardiogram-gated CT of the aortic root and annulus, and a non-gated CT of the chest, abdomen, and pelvis [24]. Coronary angiography is recommended due to the high incidence of coronary artery disease in patients undergoing TAVR (40% to 75%). The long-term clinical benefits of elective revascularization before TAVR are unclear at this time and not routinely performed [25]. A multidetector CT (MDCT) is the current standard for aortic valve evaluation, providing information concerning annular sizing, aortic root sizing, and procedure planning. For sufficient visualization using this method, 80 mL to 120 mL of low-osmolar iodinated contrast is typically utilized, carrying a significant risk for nephrotoxicity. In patients in whom iodinated contrast is contraindicated, alternative imaging including a transesophageal echocardiogram (TEE) for valve sizing and MRI for vascular access, are recommended, but these modalities are highly dependent on local expertise and more often require multimodality integration.

Lastly, another CT imaging involving the major thoracic arterial system, carotids, thoracoabdominal aorta, and iliofemoral vasculature, is recommended for the planning of vascular access [24].

High Risk of AKI With Standard Pre-TAVR Workup

Conventionally, contrast-induced nephropathy (CIN) is defined as a serum creatinine increase of >25% from baseline (>0.5 mg/dL) in the 48 to 72 hours following the procedure with additional sources of renal dysfunction having been ruled out. The exact mechanism of CIN is unclear; although, it is thought to be a combination of direct renal tubular toxicity and renal medullary hypoxia due to increased perivascular hydrostatic pressure along with direct tubular obstruction [26]. Contrast-induced nephropathy is known to be associated with contrast CT imaging and coronary angiography independently, and the need for multiple contrast-exposure events to evaluate the peripheral vasculature, coronary structure, and replacement valve apparatus sizing, inevitably increases that risk. Diabetes mellitus or atrial fibrillation, both common comorbidity in older patients with cardiovascular issues, can also increase the risk of CIN in such patients [27]. The risk of renal function impairment associated with any iodinated contrast using radiological procedures in the general population is relatively low at 0.6% to 2.3 %. However, it can be significantly high in selected patient subsets (up to 20%), mainly in patients with underlying cardiovascular disease and CKD [28]. Some studies have even claimed that it can be as high as 50% in high-risk patients with co-morbidities as mentioned above [29]. Therefore, in CKD patients requiring pre-operative evaluation for TAVR, the risk of CIN is of particular concern due to the increased risk of CIN in patients with pre-existing CKD, advanced age, and other relevant comorbidities.

## Materials and methods

A proposed protocol for CKD patients requiring evaluation for TAVR

We utilized, in our single-center study approved by the Centra Health Institute Review Board (approval no. CHIRB0415), a new protocol for patients undergoing pre-operative evaluation for TAVR. This protocol minimizes contrast exposure in CKD patients requiring TAVR by means of two contrast-sparing steps: pre-operative contrast-sparing evaluation and intra-operative contrast minimization.

**Figure 1:**
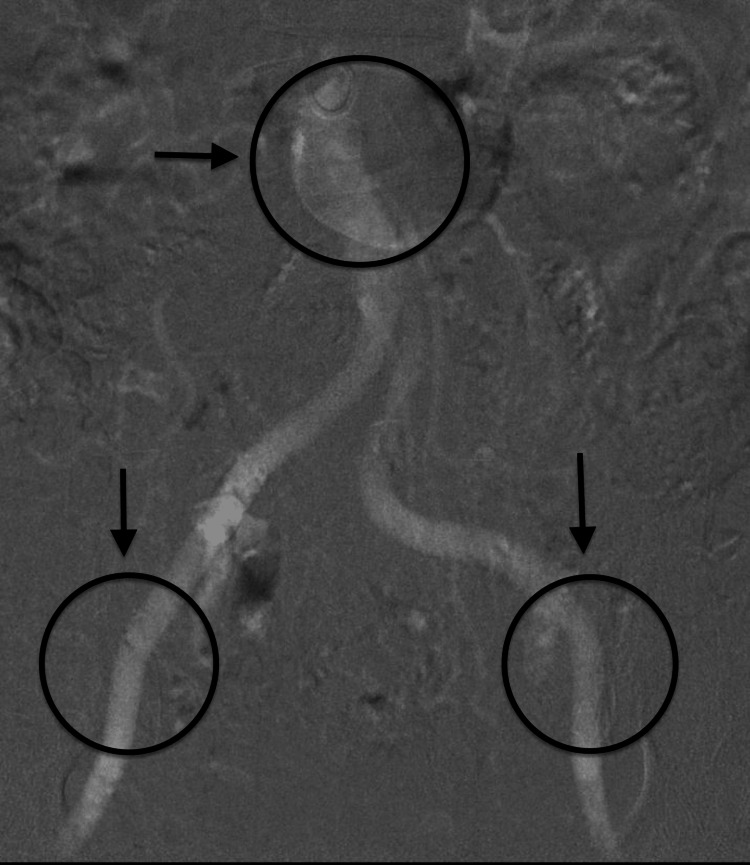
Arrows showing patent blood circulation through aortic branches

**Figure 2:**
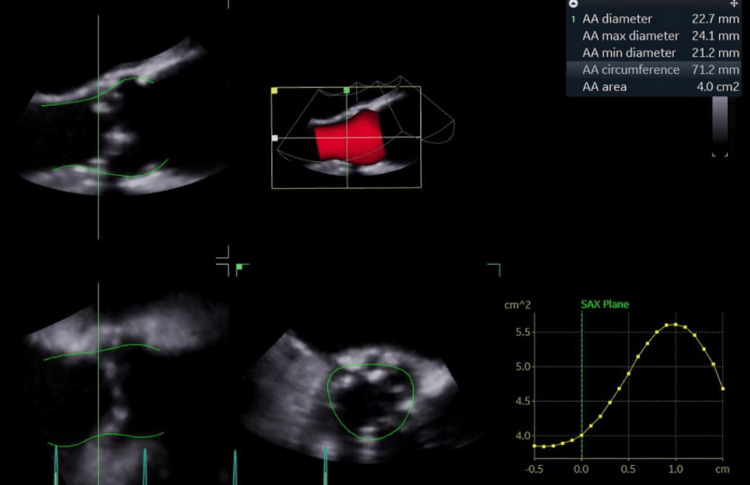
Transesophageal echocardiogram TAVR Sizing TAVR: Transcatheter aortic valve replacement

The preoperative step included limited coronary angiography, a non-contrast gated CT/MRI of the thoracic aorta, and a carbon dioxide (CO_2_) angiogram of the iliac vessels and abdominal aorta (below the level of the diaphragm) (Figure 1) with three-dimensional (3D) trans-esophageal echocardiogram (TEE) for valve sizing (Figure 2).

In several cases, the peripheral vascular access exam was supplemented with intravascular ultrasound (IVUS). The recommended standard evaluation was followed and included the following: pulmonary function testing, carotid doppler, and preoperative dental examination.

The intraoperative step utilized a two-pigtail method with placement in the non-coronary and left coronary cusps. This was used to obtain the coplanar angle and determine valve positioning. A recapturable Evolut R (Medtronic Inc., Minneapolis, MN, USA) valve was then deployed under TEE guidance with minimal use of fluoroscopy.

The other standard protocol which was followed included holding metformin, angiotensin-converting enzyme, and angiotensin receptor blocker on the day of the procedure and the following day. Unless the patient has a very low ejection fraction (like our patient in Case 1), as per American Heart Association recommendations, pre-hydration with isotonic normal saline at 1 ml/kg/hr was done for three hours before and for six hours after the procedure [30].

The following eight cases include patients with symptomatic, severe AS. These patients required TAVR while having CKD stage III (GFR 30-60) or IV (GFR 15-30). The cut-off for GFR was 15. The post-procedural outcomes of renal function and aortic valve hemodynamics are discussed. Albeit the number of cases being low for any type of study, a well-researched study has demonstrated that the number needed to treat (NNT) in TAVR literature is five only as per the landmark PARTNER trial [17]. Therefore, our cohort is of reasonable size as proof of concept to evaluate the idea of a contrast frugal approach in CKD patients to minimize kidney injury for TAVR. Note, this is a proof of concept only and we suggest that randomized controlled trials with larger cohorts be conducted in the future. 

In order to measure clinical improvement or compare pre-TAVR versus post-TAVR status, we decided to look into validated clinical parameters. We checked the New York Heart Association (NYHA) functional classification for symptom improvement and the Kansas City Cardiomyopathy Questionnaire (KCCQ) for assessment of the quality of life. Mean AS gradient pre and post-TAVR by means of transthoracic echocardiogram (TTE) demonstrated objective evidence of the degree of transvalvular flow improvement. Paravalvular aortic regurgitation was measured with TTE postoperatively to ascertain the valvular integrity and optimum placement. Left ventricular ejection fraction (LVEF) was measured pre and post-TAVR as a marker of overall or global cardiac function. When mentioned as a range (eg., 50% to 60%), the middle point of the range was taken as the LVEF (eg., 55%). All patients had eGFR calculated based on their age, ethnicity, and serum creatinine before TAVR and on a follow-up about one month and one year postoperatively in order to document the baseline renal function and short-term or long-term impact of this TAVR protocol on the prognosis of kidney function in patients with advanced CKD. In our cohort, there was zero contrast intraoperatively, CO_2_ angiography was performed for the iliofemoral system, and IVUS was used for trans-axillary access (when needed). Preoperatively, non-contrast planning CT, TEE, and CO_2_ angiography of the iliofemoral system were used. Coronary angiography was performed using less than 20 ccs of contrast in every case. 

## Results

Our pilot study enrolled a total of eight patients. Basic demographic and clinical information about the participants is presented in Table 1.

**Table 1:** Patient characteristics eGFR: estimated glomerular filtration rate; BSA: Body surface area; CABG: Coronary artery bypass grafting

Characteristics	Indices	Numbers ( percentage)
Age		73.75 ± 10.39
Sex	Female	2 (25%)
Race	Black	1 (12.5%)
Baseline eGFR	ml/min/1.73 m^2 ^BSA	27.38 ± 8.72
Diabetes mellitus	Yes	5 (62.5%)
Previous CABG	Yes	1 (12.5%)
Previous pacemaker	Yes	2 (25%)
Smoking history	Current tobacco smoker	None
Hypertension	Yes	8 (100%)
Atrial fibrillation	Yes	4 (50%)

All except one patient were >65 years old and the mean age of the group was about 74. The participants were predominantly male (75%) and Caucasian (87.5%) with a significant prevalence of diabetes mellitus (62.5%) and hypertension (100%). None of the patients was an active current smoker. There were two patients with previous permanent pacemakers and one with a history of coronary artery bypass graft (CABG) surgery. Half of the patients had atrial fibrillation. The majority of our participants (75%) had advanced CKD stage 4 with eGFR between 15 and 29 ml/min/per 1.73 m^2^ body surface area (BSA) and two participants were CKD stage 3B or close (eGFR 30-45 ml/min/per 1.73 m^2^ BSA). The eGFR was calculated based on modification of diet in renal disease (MDRD) calculation used to measure renal function.

The New York Heart Association functional classification demonstrated statistically significant improvement in symptomatology (p=0.0001) by the one-year mark post-TAVR. Although the KCCQ scores were noted to be overall improved pre to post-TAVR, the numerical improvement did not reach statistical significance to suggest a definite improvement in quality of life. As would be expected, patients’ mean aortic stenosis gradient was significantly improved (p=0.00004) after the TAVR procedure. No significant post-operative paravalvular aortic regurgitation was noted on follow-up TTE. There was no statistically significant difference noted in LVEF when comparing pre and postoperative TTE data. The eGFR data showed no clear pattern in relation to the TAVR procedure, however, the mean eGFR for the group was slightly better (27.38 ml/min/per 1.73 m^2^ BSA Vs 30.38 ml/min/per 1.73 m2 BSA) after TAVR. This observation was not statistically significant. The cardiovascular and renal outcomes pre and post-TAVR are outlined in Table 2.

**Table 2:** Cardiovascular and renal parameters pre and post-TAVR procedure NYHA: New York Heart Association; TAVR: Transcatheter aortic valve replacement; LVEF: Left ventricular ejection fraction; KCCQ: Kansas City Cardiomyopathy Questionnaire; eGFR: Estimated glomerular filtration rate; N/A: Not applicable

Participants' parameters			1	2	3	4	5	6	7	8	p-value (Pre-TAVR vs 1 year)
Values	NYHA level	Pre-TAVR	3	3	3	3	3	3	3	3	0.0001
		Post-1 year	1	2	2	2	2	2	1	1	
	LVEF	Pre-TAVR	25	65	35	60	65	60	60	65	0.26
		Post- 1 month	55	65	35	60	55	60	60	65	
		Post- 1 year	55	65	55	60	55	60	65	65	
	Mean Aortic Stenosis Gradient	Pre-TAVR	32	66	48	49	45	40	44	56	0.00004
		Post- 1 month	8	14	8	5	18	8	25	9	
		Post- 1 year	N/A	18	9	9	18	13	18	14	
	Paravalvular Regurgitation (0-4)	Post- 1 month	0	1	0	0	1	0	0	1	
	KCCQ	Pre-TAVR	N/A	54	33	25	17	31	42	N/A	0.13
		Post- 1 year	N/A	57	34	62	31	38	N/A	41	
	eGFR	Pre-TAVR	18	24	31	28	26	27	46	19	0.45
		Post- 1 month	19	15	40	37	32	27	50	16	
		Post- 1 year	19	9	48	26	23	39	59	20	

## Discussion

Our goal is to show that our “minimal-IV dye” protocol yields the same expected cardiac outcome (LVEF, paravalvular leak, KCCQ) but preserved or ensured a better renal outcome (no statistically significant decline in eGFR and rather better eGFR post-TAVR due to postulated better cardiac output after valve surgery). The results of our pilot study are summarized in Table 3.

**Table 3:** Study highlights LVEF: Left Ventricular Ejection Fraction; TAVR: Transcatheter aortic valve replacement; TAVT: Transcatheter aortic valve replacement ' eGFR: estimated glomerular filtration rate; NYHA: New York Heart Association

	Salient findings
1	The “contrast frugal” approach is feasible and safe. There was no significant paravalvular leak or misplacement of the prosthetic valve following this proposed protocol of pre-procedure evaluation.
2	There was a statistically significant improvement in symptoms by one-year post-TAVR (as per the NYHA classification)
3	Slightly better mean eGFR in the post-TAVR period on one-year follow-up (though not statistically significant)
4	No statistical improvement in LVEF in the pre and post-TAVR group
5	The mean aortic stenosis gradient improved significantly after the procedure

As we have described earlier, TAVR is expected to be more prevalent in the future and it is currently considered the standard of care for more and more patients, including those with CKD. This approach will help preserve renal function, especially for those with advanced CKD (stages 3b, 4, and 5), and at the same time allow the very essential heart-saving surgery for better patient outcomes. Although iso-osmolar or low osmolar dye is postulated to be better choices for renal protection, it still carries a significant risk of CIN [31]. It is known that mortality rates are higher in patients requiring new dialysis after TAVR [23]. In our pilot study, we demonstrated that by lowering the contrast exposure, we would not compromise the cardiovascular outcome of the patients while maintaining the integrity of renal function, one year after the procedure. Our minimal dye exposure technique could be applied to a larger population of CKD patients in an attempt to prevent CKD progression and end-stage renal disease. The medicare expenses of the USA for renal failure patients are significantly high already [32], and by mitigating the risk factors of CIN for TAVR, we could lower the cost burden of dialysis patients and attempt to reduce the huge financial expenses incurred from the premature need for dialysis.

Study limitations

Our patient population was very small and included only eight patients which is not an ideal representation of the CKD population. The improvement in eGFR cannot be concluded for certain in the general population unless a much bigger study is conducted. The age of the patients was older and does not represent the younger population. Due to decreased sample size, we could not gather enough female patients or other racial background populations hence this study outcome needs to be tested in a more diverse population setting through a larger multi-center study. The follow-up period was limited to only one year and a longer follow-up is needed to assess long-term renal outcomes.

## Conclusions

The "contrast frugal” approach is feasible and safe for pre-TAVR evaluation and the procedure itself. Our pilot study showed no significant paravalvular leak of the prosthetic valve following this proposed protocol. No statistically significant decrease in eGFR was noted on a one-year follow-up. This could be a safer option in moderate to severe aortic stenosis patients at risk of worsening renal function. More studies need to be done on this approach to help preserve kidney function in the long run.
